# Study of the short-term quality of life of patients with esophageal cancer after inflatable videoassisted mediastinoscopic transhiatal esophagectomy

**DOI:** 10.3389/fsurg.2022.981576

**Published:** 2023-01-06

**Authors:** Gaoxiang Wang, Xiaohui Sun, Tian Li, Meiqing Xu, Mingfa Guo, Changqing Liu, Mingran Xie

**Affiliations:** Department of Thoracic Surgery, The First Affiliated Hospital of USTC, Division of Life Sciences and Medicine, University of Science and Technology of China, Hefei, China

**Keywords:** esophageal cancer, minimally invasive esophagectomy, mediastinoscopy, QLQ-C30, surgery

## Abstract

**Objective:**

To compare the short-term outcomes and postoperative quality of life in patients with esophageal cancer between inflatable videoasisted mediastinoscopic transhiatal esophagectomy (IVMTE) and minimally invasive Mckeown esophagectomy (MIME), and to evaluate the value of IVMTE in the surgical treatment of esophageal cancer.

**Methods:**

A prospective, nonrandomized study was adopted. A total of 60 esophageal cancer patients after IVMTE and MIME December 2019 to January 2022 were included. Among them, 30 patients underwent IVMTE and 30 patients underwent MIME. Shortterm outcomes (including the operation time, intraoperative blood loss, postoperative drainage 3 days, total postoperative tube time, postoperative hospital stay, number and number of thoracic lymph node dissection stations, postoperative complications and so on), postoperative quality of life, [including Quality of Life Core Questionnaire (QLQ-C30) and the esophageal site-specific module (QLQ-OES18)] were compared between the 2 groups.

**Results:**

The operation time, intraoperative blood loss, postoperative drainage volume and total postoperative intubation time in IVMTE group were significantly lower than those in MIME group (*P* < 0.05). A total of 22 patients had postoperative complications, including 7 patients in IVMTE group (23.3%) and 15 patients in MIME group (50.0%). There was significant difference between the two groups (*P* = 0.032). The physical function, role function, cognitive function, emotional function and social function and the overall health status in the IVMTE group were higher than those in the MIME group at all time points after operation, while the areas of fatigue, nausea, vomiting and pain symptoms in the MIME group were lower than those in the MIME group at all time points after operation.

**Conclusion:**

IVMTE is a feasible and safe alternative to MIME. Therefore, when the case is appropriate, IVMTE should be given priority, which is conducive to postoperative recovery and improve the quality of life of patients after operation.

## Introduction

Esophageal cancer (EC) is one of the most common malignant tumors, and its mortality rate is the fourth among all malignant tumors in the world (1, 2). With the development of minimally invasive endoscopic technology, minimally invasive surgery for esophageal cancer mainly includes minimally invasive McKeown esophagectomy, minimally invasive Ivor-Lewis esophagectomy and mediastinoscopic esophagectomy (3–5). The goal of tumor surgical treatment is to pursue a higher quality of life on the basis of ensuring tumor radical resection and surgical safety (6–8). Compared with open esophagectomy, thoracic laparoscopy combined with McKeown esophagectomy can significantly improve the postoperative quality of life of patients with esophageal cancer. In addition, with the continuous development of endoscopic surgery, mediastinoscopy combined with laparoscopy has become a new method for the treatment of esophageal cancer because of its advantages such as less trauma, less postoperative complications and less postoperative pain (9, 10). However, at present, there are few studies at home and abroad on whether inflatable videoasisted mediastinoscopic transhiatal esophagectomy (IVMTE) can further improve the postoperative quality of life without affecting the efficacy and safety of the operation. In this study, a prospective non-randomized study was conducted to analyze the clinical data of 60 patients who underwent IVMTE and minimally invasive McKeown esophagectomy (MIME) in the Department of Thoracic surgery of the First Affiliated Hospital of University of Science and Technology of China from December 2019 to January 2022. The clinicopathological data, perioperative data and short-term postoperative quality of life of the two groups were compared.

## Materials and methods

### Object of study

In this study, the clinical data of patients with IVMTE and MIME in the Department of Thoracic surgery of the First Affiliated Hospital of University of Science and Technology of China from December 2019 to January 2022 were collected prospectively and non-randomly. All patients received esophageal cancer resection and lymph node dissection. The inclusion criteria were as follows: (1) Postoperative pathological diagnosis of esophageal squamous cell carcinoma, (2) MIME or IVMTE combined with laparoscopic resection of esophageal cancer, (3) Did not receive neoadjuvant therapy before operation, (4) The operation is R0 resection. The exclusion criteria were as follows: (1) Distant metastasis was found during the operation, (2) Conversion to thoracotomy during operation, (3) The case data are incomplete.

Through the inclusion and exclusion criteria, 60 patients were included, including 43 males and 17 females, with an average age of 68.17 ± 8.474 years (range: 52–83 years). Patients were informed of two surgical methods before operation. first, patients were divided into groups according to their wishes, and patients who did not express special wishes decided the surgical methods according to their own conditions: IVMTE group (*n* = 30) and MIME group (*n* = 30).

All patients underwent blood routine, biochemistry, coagulation, immunohistochemistry, esophageal barium meal, electronic gastroscope, ultrasonic endoscopy, electrocardiogram, echocardiography, pulmonary function examination, chest + upper abdominal enhanced CT, neck and abdominal B-ultrasound examination to determine the size of the tumor, the depth of invasion, the relationship with surrounding tissues, the location and size of lymph nodes, and exclude distant metastasis and obvious invasion of esophageal tumors. Some patients underwent PET-CT for preoperative staging.

AJCC eighth edition TNM staging system was used for tumor staging. All patients were evaluated according to the American Association of Anesthesiologists (ASA) classification criteria before operation. Postoperative complications were evaluated by Clavien-Dindo surgical complication grading standard. The complications of Clavien-Dindo grade 1–2 were classified as minor complications and Clavien-Dindo grade 3–5 as major complications. This study was approved by the First Affiliated Hospital of University of Science and Technology of China (O.2022-RE-145).

### Surgical procedures

IVMTE: After single-lumen endotracheal intubation anesthesia, the patient took the supine position, the shoulder and back pad was high, and the head was tilted back to the right to fully expose the left neck. A 3 cm cervical incision was made along the medial side of the left sternocleidomastoid muscle, cut layer by layer, separated the muscle group, dissociated the cervical esophagus, and marked to protect the recurrent laryngeal nerve. Place incision protective cover, jacket gloves to establish a closed cavity, mediastinal inflatable pressure 8 mmHg (1 mmHg = 0.133 kPa), flow 5 L-6/min, insert operating instruments. Dissociate downward along the left wall of the cervical esophagus in the order of “left-anterior-right posterior” to the level of subCarina or lower pulmonary ligament, and dissect the lymph nodes adjacent to the recurrent laryngeal nerve, esophagus and subCarina. Attention should be paid to the protection of recurrent laryngeal nerve, azygos vein arch and thoracic duct during operation. Abdominal operation and digestive tract reconstruction are the same as MIME.

MIME: The patient first took the left lateral decubitus and left one-lung ventilation. The fourth intercostal 2 cm incision of the right anterior axillary line was selected as the main operating hole, the 7th intercostal 1 cm incision of the midaxillary line was used as the mirror hole, and the 8th intercostal 1.5 cm incision between the posterior axillary line and the scapular line was used as the auxiliary operation hole. The azygos vein was ligated with HEMO-LOCK and then severed. Ultrasonic scalpel and electric hook were used to dissociate the thoracic esophagus. Finally, the lymph nodes adjacent to the recurrent laryngeal nerve, esophagus and Carina were routinely explored. The suspected enlarged lymph nodes were dissected during the operation, and no obvious enlarged lymph nodes were sampled. The horizontal position was taken at the end of the chest operation, and the abdominal operation was the same as that in the mediastinoscopy group. After the tube stomach was made, the incision along the medial edge of the left cervical sternocleidomastoid muscle was made, the cervical esophagus was dissociated, and the esophagus was pulled out through the cervical incision. the cervical esophagus was anastomosed end to side with a circular stapler, and after the anastomosis was completed, the gastric tube and duodenal nutrition tube were placed through the abdominal incision to close the abdominal incision and cervical incision.

### Observation indicators

The clinicopathological data and perioperative indexes were collected. The clinicopathological data included sex, age, body mass index (BMI), tumor location, pTNM stage, tumor differentiation, ASA grade and preoperative comorbidities. Perioperative indexes included operation time, intraoperative blood loss, postoperative drainage 3 days, total postoperative tube time, postoperative hospital stay, number of lymph node dissection and number of lymph node dissection stations, postoperative complications and so on.

The quality of life was evaluated by Quality of Life Core Questionnaire (QLQ-C30) and the esophageal site-specific module (QLQ-OES18) evaluation scale of European Organization for Cancer treatment and Research. The evaluation time was 1 day before operation and 1, 2, 4 and 8 weeks after operation. QLQ-C30 is divided into 15 areas and 30 projects. Items 29 and 30 contain 7 grades with a score of 1–7, while the other items have 4 grades. 1–4 points are assigned respectively. The scale included functional areas (social function, cognitive function, physical function, emotional function, role function), symptom areas (fatigue, nausea and vomiting, pain), and general health status. And 6 single items (dyspnea, insomnia, loss of appetite, constipation, diarrhea, economic hardship). Divide the sum of project scores in each area by the number of projects, that is, the rough score in that field. The higher the score of functional index and comprehensive quality of life dimension, the better the function and the higher the quality of life; the higher the score of symptomatic index, the more serious the symptom and the worse the quality of life. The scores of each subscale were calculated as follows: functional subscale: s = {1-(RS-1)/range} * 100; symptom subscale: s = {(RS-1)/range} * 100; general health status subscale: s = {(RS-1)/range} * 100. Among them, RS represents the original score, Range represents the extremely poor score, the functional subscale and symptom subscale have a very poor score of 3, while the total health subscale has a very poor score of 6. The medical staff completed all the scales through face-to-face interview or telephone follow-up.

### Statistical analyses

SPSS26.0 statistical software was used for data analysis. The normal distribution data are expressed by $\bar{X}\pm S$, the measurement data are compared by t-test, the counting data are compared by *χ*^2^ test, the skewed data are described by median M (P25-P75), and the comparison between the two groups is Wilcoxon rank sum test (statistics is Uc). Repeated measurement analysis of variance was used to compare the quality of life at different time points. *P* < 0.05 means that the difference is statistically significant.

## Result

### Comparison of clinicopathological data

There was no significant difference in sex, age, BMI, tumor location, pTNM stage, tumor differentiation, ASA grade and preoperative comorbidities between the two groups (*P* > 0.05, Table 1).

**Table 1:** Comparison of clinicopathological data between the two groups.

	IVMTE Group (*n* = 30)	MIME Group (*n* = 30)	*χ* ^2^ */t*	*P*
Sex			0.082	0.774
Male	21 (70.0%)	22 (73.3%)		
Female	9 (30.0%)	8 (26.7%)		
Age	68.60 ± 8.228	67.73 ± 8.832	0.393	0.696
BMI	21.48 ± 3.179	21.53 ± 2.823	−0.065	0.948
Tumor location			0.430	0.806
Up	3 (10.0%)	3 (10.0%)		
Medium	20 (66.7%)	22 (73.3%)		
Low	7 (23.3%)	5 (16.7%)		
pTNM stage			4.431	0.109
I	17 (56.7%)	9 (30.0%)		
II	8 (26.7%)	14 (46.7%)		
III	5 (16.7%)	7 (23.3%)		
Tumor differentiation			0.348	0.840
Low	11 (36.7%)	13 (43.3%)		
Medium	12 (40.0%)	10 (33.3%)		
High	7 (23.3%)	7 (23.3%)		
ASA grade			0.098	0.754
II	6 (20.0%)	7 (23.3%)		
III	24 (80.0%)	23 (76.7%)		
Preoperative comorbidities^a^			0.287	0.592
Yes	12 (40.0%)	10 (33.3%)		
No	18 (60.0%)	20 (66.7%)		

IVMTE, inflatable videoasisted mediastinoscopic transhiatal esophagectomy; MIME, minimally invasive McKeown esophagectomy; BMI, body mass index.

^a^ Includes high blood pressure, diabetes, arrhythmia, and so forth.

### Comparison of perioperative data

The operation time, intraoperative blood loss, postoperative drainage 3 days and total postoperative tube time in IVMTE group were significantly lower than those in MIME group (*P* < 0.05, Table 2). There was no significant difference in postoperative hospital stay between IVMTE group and MIME group (*P* > 0.05, Table 2). There was no significant difference in the number of lymph node dissection and number of lymph node dissection stations between the two groups (*P* > 0.05, Table 2).

**Table 2:** Comparisons of perioperative data between the two groups.

	IVMTE Group (*n* = 30)	MIME Group (*n* = 30)	*t/Z*	*P*
Operation time (min)	217.2 ± 38.646	264.9 ± 47.575	−4.260	<0.001
Intraoperative blood loss (ml)	50 (50,100)	100 (100,100)	−4.259	<0.001
Postoperative drainage 3 days (ml)	386.5 (244,650)	800.0 (631,883)	−4.429	<0.001
Total postoperative tube time (d)	9.0 (8,10)	9.0 (9,13)	−2.003	0.045
Postoperative hospital stay (d)	9.0 (9,11)	10.0 (9,15)	−1.199	0.230
The number of lymph node dissected station	4.5 ± 0.900	5.1 ± 1.570	−0.182	0.076
The number of lymph node dissected	14.5 ± 5.270	15.3 ± 4.748	−0.592	0.556

IVMTE, inflatable videoasisted mediastinoscopic transhiatal esophagectomy; MIME, minimally invasive McKeown esophagectomy.

### Postoperative complications

A total of 22 patients had postoperative complications, including 7 patients in IVMTE group (23.3%) and 15 patients in MIME group (50.0%). There was significant difference between the two groups (*P* = 0.032, Table 3). In addition, the incidence of pulmonary complications in the IVMTE group was lower than that in the MIME group, and the results were statistically significant (*P* < 0.05, Table 3). However, there was no significant difference in pulmonary infection, recurrent laryngeal nerve injury, incision infection and anastomotic leakage between the two groups (*P* > 0.05, Table 3).

**Table 3:** Comparisons of postoperative complications between the two groups.

	IVMTE Group (*n* = 30)	MIME Group (*n* = 30)	*χ* ^2^	*P*
Minor complications (Clavien-Dindo grade 1–2)				
Pulmonary leakage	0 (0.0%)	2 (6.7%)	0.517	0.472
Pulmonary infection	2 (6.7%)	6 (20.0%)	1.298	0.255
Recurrent laryngeal nerve injury	1 (3.3%)	3 (10.0%)	0.268	0.605
Incisional infection	1 (3.3%)	4 (13.3%)	0.873	0.350
Arrhythmia	2 (6.7%)	4 (13.3%)	0.185	0.667
Major complications (Clavien-Dindo grade 3–5)				
Pulmonary infection	1 (3.3%)	4 (13.3%)	0.873	0.350
Chylothorax	0 (0.0%)	2 (6.7%)	0.517	0.472
Anastomotic leakage	2 (6.7%)	4 (13.3%)	0.185	0.667
Reoperation	1 (3.3%)	3 (10.0%)	0.268	0.605
Postoperative complications rate	7 (23.3%)	15 (50.0%)	4.593	0.032
Pulmonary complications rate	3 (10.0%)	10 (33.3%)	4.812	0.028

IVMTE, inflatable videoasisted mediastinoscopic transhiatal esophagectomy; MIME, minimally invasive McKeown esophagectomy.

### Comparison of quality of life

**Figure 1:**
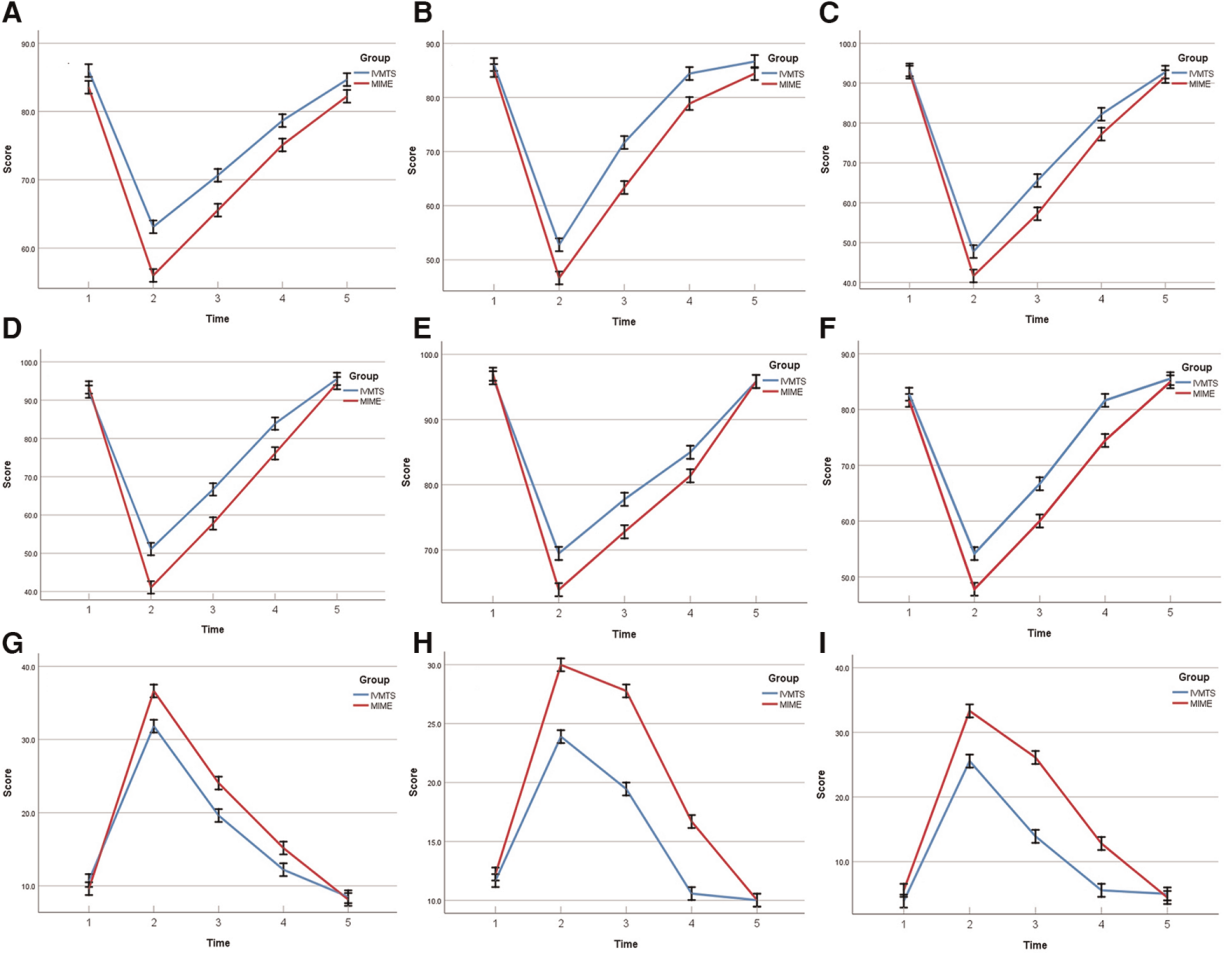
Comparison of QLQ-C30 scores between the two groups before and after operation. (**A**) Physical function. (**B**) Role function. (**C**) Cognitive function. (**D**) Social function. (**E**) Emotional function. (**F**) Global health status. (**G**) Fatigue. (**H**) Nausea/vomiting. (**I**) Pain. IVMTE, inflatable videoasisted mediastinoscopic transhiatal esophagectomy; MIME, minimally invasive Mckeown esophagectomy; Time 1, 1 day before operation; Time 2, 1 weeks after operation; Time 3, 2 weeks after operation; Time 4, 4 weeks after operation; Time 5;, 8 weeks after operation.

**Figure 2:**
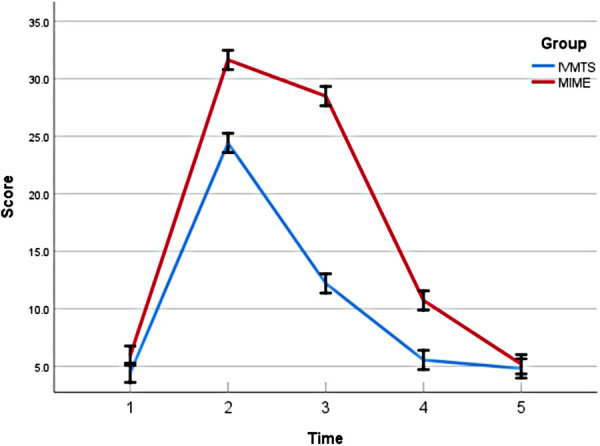
Comparison of QLQ-OES18 scores (pain) between the two groups before and after operation. IVMTE, inflatable videoasisted mediastinoscopic transhiatal esophagectomy; MIME, minimally invasive Mckeown esophagectomy; Time 1, 1 day before operation; Time 2, 1 weeks after operation; Time 3, 2 weeks after operation; Time 4, 4 weeks after operation; Time 5, 8 weeks after operation.

In terms of QLQ-C30 score, the preoperative functional domain, symptom domain and overall health scores of the two groups were similar, the functional domain and overall health scores decreased significantly 1 week after operation, and the scores increased gradually at 2, 4 and 8 weeks after operation, while the symptom domain scores increased significantly 1 week after operation, and the node scores decreased gradually at 2 weeks, 4 weeks and 8 weeks after operation. The physical function, role function, cognitive function, emotional function and social function and the overall health status in the IVMTE group were higher than those in the MIME group at all time points after operation, while the areas of fatigue, nausea, vomiting and pain symptoms in the MIME group were lower than those in the MIME group at all time points after operation (Table 4 and Figures 1A–I). In addition, the QLQ-OES18 score of the IVMTE group was lower than that of the MIME group at each time point after operation (Table 5 and Figure 2).

**Table 4:** Quality of life scores in IVMTE and MIME using QLQ-C30.

	Mean square	*F*	*P*
Physical function			
Main effect of Time factor	10,792.620	740.457	<0.001
Main effect of grouping factors	1,282.987	6.814	0.011
Time grouping interaction	94.144	6.459	0.001
Role function			
Main effect of Time factor	18,898.780	514.319	<0.001
Main effect of grouping factors	1,633.333	14.175	<0.001
Time grouping interaction	172.284	4.689	0.003
Cognitive function			
Main effect of Time factor	36,153.289	512.626	<0.001
Main effect of grouping factors	1,339.008	7.356	0.009
Time grouping interaction	229.564	3.255	0.025
Social function			
Main effect of Time factor	26,064.697	626.620	<0.001
Main effect of grouping factors	2,133.867	10.191	0.002
Time grouping interaction	374.518	9.004	<0.001
Emotional function			
Main effect of Time factor	22,700.181	852.750	<0.001
Main effect of grouping factors	556.241	10.191	0.002
Time grouping interaction	269.278	10.116	<0.001
Global health status			
Main effect of Time factor	16,474.643	210.925	<0.001
Main effect of grouping factors	1,441.459	9.507	0.003
Time grouping interaction	210.220	2.691	0.047
Fatigue			
Main effect of Time factor	7,764.444	206.436	<0.001
Main effect of grouping factors	345.399	6.512	0.013
Time grouping interaction	129.003	3.430	0.013
Nausea/vomiting			
Main effect of Time factor	4,639.294	52.418	<0.001
Main effect of grouping factors	1,332.678	8.688	0.005
Time grouping interaction	280.769	3.172	0.026
Pain			
Main effect of Time factor	8,480.382	157.072	<0.001
Main effect of grouping factors	2,402.670	16.790	<0.001
Time grouping interaction	471.112	8.726	<0.001
Dyspnea			
Main effect of Time factor	3,211.800	17.473	<0.001
Main effect of grouping factors	132.934	0.095	0.759
Time grouping interaction	178.660	0.972	0.386
Insomnia			
Main effect of Time factor	1,777.243	11.195	<0.001
Main effect of grouping factors	132.934	0.166	0.685
Time grouping interaction	44.718	0.282	0.826
Appetite loss			
Main effect of Time factor	16,531.346	61.706	<0.001
Main effect of grouping factors	371.186	0.372	0.544
Time grouping interaction	14.122	0.053	0.975
Constipation			
Main effect of Time factor	460.955	3.556	0.024
Main effect of grouping factors	14.785	0.017	0.896
Time grouping interaction	56.062	0.432	0.684
Diarrhea			
Main effect of Time factor	671.880	3.589	0.012
Main effect of grouping factors	59.141	0.228	0.635
Time grouping interaction	26.875	0.144	0.946
Financial diffculties			
Main effect of Time factor	1,404.506	6.932	0.001
Main effect of grouping factors	949.452	0.261	0.611
Time grouping interaction	112.841	0.557	0.584

IVMTE, inflatable videoasisted mediastinoscopic transhiatal esophagectomy; MIME, minimally invasive McKeown esophagectomy.

**Table 5:** Quality of life scores in IVMTE and MIME using QLQ-OES18.

	Mean square	*F*	*P*
Dysphagia			
Main effect of Time factor	6,994.808	42.635	<0.001
Main effect of grouping factors	41.070	0.071	0.791
Time grouping interaction	18.390	0.112	0.913
Eating			
Main effect of Time factor	7,207.860	43.719	<0.001
Main effect of grouping factors	180.496	0.206	0.652
Time grouping interaction	274.735	1.666	0.186
Reflux			
Main effect of Time factor	1,015.949	9.287	<0.001
Main effect of grouping factors	45.241	0.060	0.807
Time grouping interaction	54.803	0.501	0.618
Pain			
Main effect of Time factor	8,705.518	154.966	<0.001
Main effect of grouping factors	2,795.632	18.974	<0.001
Time grouping interaction	812.818	14.469	<0.001
Saliva			
Main effect of Time factor	1,197.308	10.225	<0.001
Main effect of grouping factors	14.785	0.017	0.898
Time grouping interaction	61.930	0.529	0.603
Choking			
Main effect of Time factor	6,253.319	34.721	<0.001
Main effect of grouping factors	3.696	0.005	0.943
Time grouping interaction	42.929	0.238	0.795
Dry mouth			
Main effect of Time factor	582.547	6.444	0.004
Main effect of grouping factors	92.408	0.107	0.745
Time grouping interaction	0.000	0.000	1.000
Taste			
Main effect of Time factor	7,788.131	38.451	<0.001
Main effect of grouping factors	14.956	0.021	0.884
Time grouping interaction	71.417	0.353	0.781
Cough			
Main effect of Time factor	294.550	4.764	0.009
Main effect of grouping factors	110.608	0.108	0.744
Time grouping interaction	6.315	0.102	0.910
Speech			
Main effect of Time factor	294.550	4.764	0.009
Main effect of grouping factors	103.803	0.111	0.740
Time grouping interaction	6.315	0.102	0.910

IVMTE, inflatable videoasisted mediastinoscopic transhiatal esophagectomy; MIME, minimally invasive McKeown esophagectomy.

## Discussion

With the progress of endoscopic technology, thoracic surgeons pursue minimally invasive surgery to achieve better surgical results and long-term prognosis. At the same time, we also pay attention to the impact of surgical methods on the quality of life of patients. Therefore, how to minimize the impact of surgery on patients is a hot issue concerned by thoracic surgeons. In addition, the impact of different surgical methods on the quality of life of surgical patients is unknown. The results of this study show that the same surgical effect can be achieved compared with MIME, IVMTE, and there are advantages in operation time, intraoperative blood loss, postoperative drainage 3 days and total postoperative tube time. In addition, in terms of postoperative complications, IVMTE is superior to MIME in the overall incidence of complications and the incidence of pulmonary complications. In the study of quality of life, IVMTE has advantages in many functional dimensions, such as physical function, role function and cognitive function, as well as in the overall health status and symptoms such as fatigue, nausea, vomiting and pain.

The pursuit of more minimally invasive surgery on the premise of ensuring the scope of oncology resection has always been the goal of thoracic surgeons. IVMTE can achieve or even better than the surgical effect of MIME and the range of lymph node dissection. In this study, it was found that IVMTE had shorter operation time, less intraoperative blood loss, less drainage 3 days after operation and shorter total time with catheter than MIME. In addition, there was no significant difference between IVMTE and MIME in the number of lymph node dissection stations and enumeration. Jin et al. (11) through a retrospective analysis of 30 MIME patients and 19 IVMTE patients, found that compared with MIME patients, IVMTE patients had shorter average operation time, less intraoperative blood loss, less drainage in the first 3 days after operation and less hospital stay. In addition, Jin et al. (11) found that there was no significant difference in the number and total number of lymph node dissection between the two groups. However, dissection of the right posterior recurrent lymph nodes in the MIME group was more common. Shi et al. (12) found that the operation time, intraoperative blood loss and postoperative hospital stay in IVMTE group were shorter than those in IVMTE group. We analyze the reasons for this result may be as follows: first, there is no need to change the position during the operation, only one position can be performed on the neck and abdomen, and the thoracic and abdominal operations can be performed at the same time, which greatly shortens the operation time and anesthesia time. Second, by inflating the mediastinum and endoscopic magnification, the anatomical structure around the esophagus can be clearly identified and the downstream from the esophagus can be seen, which avoids the blindness of the traditional esophagectomy and effectively reduces the injury of nerves, blood vessels and thoracic ducts during the operation. reduce the risk of surgical bleeding and postoperative tissue exudation. Third, there is no need to place thoracic drainage tube after operation and does not cause damage to the intercostal nerve, reduce postoperative drainage and postoperative pain, make patients get out of bed earlier and accelerate their recovery.

Postoperative complications have always been one of the key factors affecting the quality of life of patients after operation. According to the study of postoperative complications of the two surgical methods, it was found that the total incidence of postoperative complications and the incidence of pulmonary complications in IVMTE group were better than those in MIME group. However, there was no significant difference in postoperative complications such as pulmonary air leakage, pulmonary infection and recurrent laryngeal nerve injury between the two groups. Rezaei et al. (13) through the study of 31 cases of IVMTE and 31 cases of MIME esophageal cancer, found that the incidence of early postoperative complications and postoperative cardiopulmonary complications in IVMTE was lower than that in MIME. Chen et al. (14) found that the incidence of postoperative pulmonary complications in IVMTE was lower than that in MIME through retrospective analysis of propensity matching. We analyze the following possible reasons for this result: first, the failure to choose the transthoracic approach reduces the possible mechanical damage to the heart, lung and other important organs during the operation, and reduces the probability of cardiovascular and pulmonary complications. Second, it avoids intercostal nerve injury and reduces postoperative pain, which is beneficial to patients’ effective cough and expectoration, and promotes the reexpansion of the lung and the oxygenation state of the patients. Third, there is no need to cut off azygos vein and bronchial artery during operation, which avoids liver function injury in some patients with liver insufficiency and reduces the probability of postoperative cough.

With the continuous improvement of social material living standards, while treating diseases, postoperative quality of life has also become an important index for doctors and patients to pay attention to. In this study, we used the QLQ-C30 and QLQ-OES18 scales of the European Organization for Cancer treatment and Research to evaluate the quality of life. The results showed that there was no difference in the scores of preoperative functional areas, symptom areas and overall health status between the two groups, but there were significant advantages in multiple quality of life dimensions between the IVMTE group and the MIME group. The above results show that IVMTE has less influence on all dimensions of postoperative quality of life of patients with esophageal cancer than MIME. On the other hand, it also reflects the advantage of less trauma and faster recovery of IVMTE in patients with esophageal cancer. Through a prospective non-randomized study, Ma et al. (15) found that the scores of emotional function and overall health scale of QLQ-C30 in MIE-SM group were significantly higher than those in MIE-MC group, while the pain score in MIE-SM group was significantly lower than that in MIE-MC group. QLQ-OES18 results showed that the pain score in MIE-SM group was significantly lower than that in MIE-MC group. We believe that the main reasons are: first, because the IVMTE operation time is shorter, the intraoperative blood loss is less and the total postoperative intubation time is shorter, patients can do physical function exercise and receive nutritional support earlier, so that they can return to normal life and work more quickly. Second, IVMTE avoids intercostal nerve injury, lightens postoperative pain, reduces postoperative discomfort, reduces negative emotions, and helps to improve postoperative quality of life. Third, the incidence of postoperative complications in IVMTE group is lower, so that patients can recover their life and work status earlier. In addition, low postoperative complications can reduce patients’ fear of surgery and contribute to the improvement of postoperative quality of life.

This study has the following shortcomings: first, this study is a single-center prospective non-random study, the sample size is relatively small, may have a little bias to the results. Second, this study did not carry out long-term follow-up of the two groups of patients, and whether the two surgical methods can achieve the same long-term prognosis has not been studied. Thirdly, postoperative radiotherapy and chemotherapy may affect the evaluation of postoperative quality of life.

## Conclusion

IVMTE can reduce intraoperative bleeding, shorten operation time and reduce postoperative complications, and improve the short-term postoperative quality of life of patients with esophageal cancer. IVMTE is a feasible and safe alternative to MIME. Therefore, when the case is appropriate, IVMTE should be given priority, which is conducive to postoperative recovery and improve the quality of life of patients after operation.

## Data Availability

The original contributions presented in the study are included in the article/Supplementary Material, further inquiries can be directed to the corresponding author/s.
